# Dies, Etc.

**Published:** 1884-01

**Authors:** 

**Affiliations:** Chicago.


ARTICLE IV.

### DIES, ETC.

BY HASKELL, CHICAGO.

In the June number of the Journal appeared a "critical" reply to an article of mine written for the benefit of the younger members of the profession, and especiaely new beginners, and in which I gave the results of a long and exclusive experience in mechanical dentistry.

My object in doing this was to enable them to accomplish certain results by more simple and expedious methods than those usually employed.

It so happened that our critic having a large number of young men under his instruction, some of whom are fully tested these methods with complete satisfaction, fearing his charge would become demoralized, attempted to refute my position.

The result has been a rehash of the old twaddle about the expansion of "hydrated calcium sulphate" (we use plaster of Paris !) and the necessity of a shrinkage metal to counteract the effect, etc., etc.

Now, we don't deny the correctness of the *theory*, but do say that *practically* it doesn't amount to anything, and that facts proved by *experience* are worth infinitely more than facts proved by *theory*. This is just what we have done, and desire to impress upon the minds of the young men in the profession.

Of course "Ephraim is joined to his idols," and evidently don't wish to get out of the old rut. It is difficult to "teach old dogs new tricks," and we don't propose to try, but in a few words will reply to his statements.

As to the plaster, the expansion of the cast, in a measure counterbalances the expansion of the impression; but suppose it does not, the expansion practically amounts to nothing, and the less time our critic devotes to measuring plaster expansion, and devotes it to something more practical, the better for all concerned.

Then, again, this talk about the necessity of a *shrinking* metal for dies. I challenge any one to produce anything like as satisfactory results as to fit of plates in any given one hundred cases, as by the use of Babbit metal, to say nothing of the greater simplicity of the method; and I think I *know* when a plate is adapted to the gums and palate. I am no novice in this business, and know whereof I affirm. Furthermore, I will assert that any one who will give it a thorough trial will have no further use for zinc.

A dentist of twenty-five years experience, who did a large amount of metal work, and whom I instructed in my methods, said, after a month's experience: It makes me mad when I think of the annoyance I have had in the use of zinc all these years, when there was a metal so much better."

"It is the exception rather than the rule," that a plate needs change from the die, in fitting to the mouth, rather than *vice versa*, as our critic asserts; and I pity the student who starts out in practice with the idea that he must fit his plates to the mouth with a pair of pliers.

Then he says: "Does he mean to convey the idea that there is any possible difference in the manipulation of zinc and the alloy he advocates, save that of the somewhat increased melting point of zinc?"

I mean to say that, taking the whole process of preparing models, moulding in oiled sand, using the Babbitt metal for a die, and in swaging of the plate, there is the greatest possible difference in practical results, being *shorter*, *easier* and *surer;* and that is just *what* I mean by "in view of such results."

It is always possible "to wipe off the metal from an oiled plate;" and the man who is not capable of doing it would better quit making metal plates.

We reaffirm our statement, that it is better to "cut and lap" a full plate; for while there is no possible objection to it, as it can readily be finished so as not to show. On the other hand, it saves time and annoyance, and *does* strengthen the plate one hundred per cent. at the place where it most often breaks. This advice is not for the " lazy man," but for the busy man, who, while doing his work well has no time to *pudder.*

As to the use of asbestos, we use it when desirable, and sand where it is best.

Plate No. 28, " standard " guage, is the thickness generally used for full or partial plates, and is thick enough, and partial plates should always be doubled around the weak points. No. 24 is thick enough for backings. We burr the surface of the backing after soldering, because it leaves a better surface

Yes, " the alloy known as Babbitt metal, has its use to serve," you truly remark, and its use is of vast importance to one who does not wish to fit plates to the mouth with pliers (and we are told of some who carry a pair in almost every pocket ?) but prefer *certain* results from simple methods.

Continue to " chew the pudding string." We prefer to test the pudding on its merits.

---

